# Are Mediators of Grief Reactions Better Predictors Than Risk Factors? A Study Testing the Role of Satisfaction With Rituals, Perceived Social Support, and Coping Strategies

**DOI:** 10.1177/10541373231191316

**Published:** 2023-07-30

**Authors:** Jacques Cherblanc, Emmanuelle Zech, Susan Cadell, Isabelle Côté, Camille Boever, Manuel Fernández-Alcántara, Christiane Bergeron-Leclerc, Danielle Maltais, Geneviève Gauthier, Chantal Verdon, Josée Grenier, Chantale Simard

**Affiliations:** 1 Département des sciences humaines et sociales, 14661 Université du Québec à Chicoutimi (UQAC), Saguenay, Canada; 2 Institut de recherche en sciences psychologiques, 83415 Université Catholique de Louvain, Louvain-la-Neuve, Belgium; 3 School of Social Work, Renison University College, 8430 University of Waterloo, Canada; 4 Departamento de Psicología de la Salud, 16718 University of Alicante, Spain; 5 Département des sciences infirmières, 59310 Université du Québec en Outaouais, Gatineau, Canada; 6 Département de Travail Social, 59310 Université du Québec en Outaouais, Gatineau, Canada

**Keywords:** complicated grief, mediating factors, coping strategies, COVID-19, adults and death

## Abstract

The present study aimed to assess the mediating role of adjustment processes in known risk factors associated with prolonged grief disorder. Data were collected in March–April 2021 through an online survey of 542 Canadian adults bereaved since March 2020. The mediating role of satisfaction with funeral rituals, bereavement support, and coping strategies on grief outcomes was tested using structural equation modeling. Results showed that such adjustment processes played a significant role in the grief process and that they were better predictors than risk factors alone. Since they are more amenable determinants of grief reactions, they should be further studied using a longitudinal design.

## Introduction

The death of a significant person can provoke intense emotional reactions in the bereaved person. However, most bereaved people will be resilient (e.g., Bonanno et al., 2008), and face this event with available social or personal support (Wittouck et al., 2011). The reactions will typically ease or resolve during the first six months (World Health Organisation [WHO], 2018) or one year (American Psychiatric Association, 2022) after the loss. However, a meta-analysis reported that a proportion (9.8%) of bereaved adults will experience difficulties in grief, particularly prolonged grief disorder (PGD; Lundorff et al., 2017). In cases of traumatic grief or death associated with unexpected or unnatural causes, the percentage of PGD can increase to 49% (Djelantik et al., 2020). PGD was “approved for inclusion in the DSM-5-TR (Prigerson et al., 2021), [and is] characterized by a persistent yearning for and/or preoccupation with thoughts of the deceased and associated distressing and disabling grief symptoms” (Gang et al., 2022). The context of the COVID-19 pandemic and the specific circumstances of COVID deaths may have altered the end-of-life experience of individuals and their families (Vachon et al., 2020) as well as the conditions under which bereaved individuals could perform rituals, receive social support, as well as cope with the death.

Previously published studies suggested high levels of dysfunctional grief during the pandemic (Bovero et al., 2022; Breen et al., 2022; Lee & Neimeyer, 2022; Neimeyer & Lee, 2022; Rawlings et al., 2022). Elevated rates of (probable) PGD (27–38%) were also found among those bereaved due to COVID-19 or COVID-19-related complications in China (Tang & Xiang, 2021) and even reached a probable prevalence of 66.53% in a self-selected sample in the United States of America (Gang et al., 2022). While these studies started to examine potential predictors or risk factors for complications in grief reactions, and despite the early suggestion by Stroebe and Schut (2021) to test a full theory-based model that would include several protective or risk factors together as well as mediators of grief reactions, most of them examined predictors of PGD one by one.

Several risk factors for developing grief disorders are well-known following a death occurring in a nonpandemic or disaster context: an unexpected or violent death, death of a child or spouse, close or dependent relationship to the deceased person, social isolation or loss of a support system or friendships, history of depression, separation anxiety or post-traumatic stress disorder, traumatic childhood experiences, such as abuse or neglect, and other major life stressors, such as major financial hardships (Lobb et al., 2010; Mason et al., 2020; Mayo Clinic, 2021; Stroebe et al., 2006; Vedder et al., 2022). In addition, particular risk factors generated by the pandemic context have been identified and include, among others, the suddenness of death, the lack of opportunity for people to support the dying person at end-of-life, the impossibility for them to say the last goodbye to the person, cancelation or postponement of funeral rituals, and social isolation (Bauld et al., 2021; Beneria et al., 2021; Hernández-Fernández & Meneses-Falcón, 2021). The inability to communicate or be with someone at the end of life may cause emotional distress and anxiety (Bansal et al., 2020). For funeral practices, a review conducted by Burrell and Selman (2022) highlights the importance of meaningful and supportive memorial services for the bereaved.

It is commonly acknowledged that each person will react differently when facing the death of someone. It is essential to investigate the mediating processes explaining why bereaved individuals would develop more severe or dysfunctional grief reactions. This is particularly true when people face additional hampering conditions to their usual grief processes such as those present during the pandemic. First, the coping strategies that people develop in the face of adversity typically represent such mediators (e.g., Lazarus & Folkman, 1984). In the context of bereavement, the dual-process model (DPM) of coping with Bereavement (Stroebe & Schut, 1999) specifically addresses two types of stressors that bereaved people may face, along with their relative coping strategies. In this model, the authors identified loss-oriented stressors, which directly relate to the loss of the deceased, and restoration-oriented stressors, which relate to the changes that occur after the loss. To address these stressors, the bereaved will use different coping strategies, identified as confrontation and avoidance. Adjusting to bereavement is a dynamic process where the person will alternate between loss- and restoration-oriented stressors and between confrontation and avoidant coping strategies, what is called oscillation. The exclusive focus on one process at the expense of the other is expected to be associated with less favorable outcomes while, on the contrary, a balanced use of coping strategies will be beneficial, in line with general coping theories (e.g., Lazarus & Folkman, 1984; Suls & Fletcher, 1985). Ryckebosch-Dayez et al. (2016) showed that both confrontation and avoidant strategies were effective in reducing short-term psychological distress experienced when facing a stressor, but avoidant strategies were significantly more effective than confrontation strategies in the short term. However, this study did not address the mediating effect of coping strategies on grief reactions. Before the pandemic, another study addressed this relationship and found that less negative appraisal of bereavement-related stressors, as well as higher use of restoration-oriented strategies, mediated the link between attachment avoidance and low severity of grief reactions among bereaved individuals who had lost a romantic partner (Delespaux et al., 2013).

In the context of the pandemic, the interruption of pre- and post-mortem rituals forced people to react or cope with grief in different ways. Some studies have shown that while individuals seem not to have performed any rituals because of restrictive measures, others have created new ones or have chosen to postpone them (Cherblanc, Zech et al., 2022; Fernández & González-González, 2020; Mitima-Verloop et al., 2022). One could assume that beyond the permission or ability to perform a ritual, the individual satisfaction with the rites performed would have more impact on the adaptation to the loss. However, only a few studies examined the effect of satisfaction with rituals on grief reactions. Two studies conducted before the pandemic showed a nonsignificant relationship between satisfaction with funeral rituals and grief reactions (Birrell et al., 2020; Mitima-Verloop et al., 2021). However, most of their respondents reported positive satisfaction with the rites and showed low to moderate levels of grief reactions. A recent study showed a significant and negative association between general funeral evaluation and the presence of prolonged grief symptoms during the pandemic (Mitima-Verloop et al., 2022).

The present study aimed to assess the role of coping strategies, bereavement support, and the satisfaction level regarding performed rituals as potential mediators of risk factors associated with grief complications in the context of the COVID-19 pandemic. In line with the DPM, we hypothesized that coping strategies would play a mediating role between all risk factors and the degree of grief complications. We expected that the satisfaction level with the rituals performed would specifically mediate the link between the number of rituals prevented by the health restrictions and grief complications, and would have a negative relationship with PGD. Finally, we hypothesized that reaching out for bereavement support would have a positive relationship with living alone, but a potential negative relationship with grief complications.

## Method

### Procedure and Participants

Data were collected via an online survey on the LimeSurvey platform from a convenience sample of French-speaking Canadian residents. Multiple strategies were used to recruit participants, including a website, social media (Facebook and Instagram), television and radio interviews performed by the principal investigator, and direct mailing from the Quebec Funeral Cooperative Federation *(Fédération des coopératives funéraires du Québec*). The inclusion criteria were (1) being 18 years old and over and (2) having experienced the death of someone since the beginning of the pandemic in March 2020. To be included in the analysis, participants must have answered all questions related to the theoretical model of grief tested in the present study. From the 955 respondents who completed the survey, 542 participants had all the required data and were thus included in the study. The included participants were recruited from March 12th until April 26th, 2021.

Most of the 542 respondents included in the present analysis were from the province of Quebec (*n* = 533) and were white (not Indigenous or racialized; *n* = 529). The 542 participants were not different from those who were not included in terms of age (49.9 years vs. 49.2 years), number of days since the loss (206 days vs. 201 days), and gender (W:M 88%:12% vs. 86%:14%) (*p* > .05). Table 1 presents the sociodemographic characteristics of the participants for the total sample (*n* = 542), as well as for the subsamples of those who reached (*n* = 152) or not (*n* = 390) the cutoff score of 59 required for the diagnosis of PGD.

**Table 1.** Sociodemographic and Loss-Related Characteristics of the Samples.

Demographic characteristics	Total sample (*n* = 542)	TGI-SR score		*p*-value
		<59 (*n* = 390)	≥59 (*n* = 152)	
Gender (*n* (%))				
*Participants*				
Men	66 (12.2)	49 (12.6)	17 (11.2)	.659
Women	476 (87.8)	341 (87.4)	135 (88.8)	
*Deceased*				
Men	264 (48.7)	178 (45.6)	86 (56.6)	.063
Women	277 (51.1)	211 (54.1)	66 (43.4)	
Other	1 (0.2)	1 (0.3)	0	
Age (years; *mean* [*S*D])				
Participants	49.9 (13.2)	50.1 (13.5)	49.3 (12.6)	.443
Deceased	72.1 (18.7)	75.9 (15.8)	62.1 (21.8)	<.001
Marital status (*n* [%])				
With partner	367 (67.7)	283 (72.5)	84 (55.3)	<.001
Single	70 (12.9)	45 (11.5)	25 (16.4)	
Divorced/separated	41 (7.6)	24 (6.1)	18 (11.1)	
Widowed	64 (11.8)	38 (9.7)	26 (17.1)	
Living alone (*n* [%])				
Yes	134 (24.7)	78 (20.0)	56 (36.8)	<.001
No	408 (75.3)	312 (80.0)	96 (63.2)	
Family income (*n* [%])				
Less than 15,000$	10 (1.8)	5 (1.3)	5 (3.3)	.014
15,000$–24,999$	35 (6.5)	18 (4.6)	17 (11.2)	
25,000$–49,999$	93 (17.2)	67 (17.2)	26 (17.1)	
50,000$–99,999$	210 (38.7)	148 (37.9)	62 (40.8)	
100,000$ and over	188 (34.7)	148 (37.9)	40 (26.3)	
Missing	6 (1.1)	4 (1.0)	2 (1.3)	
Time since the loss (days; *mean* [*S*D])	205.7 (122.9)	199.0 (123.0)	222.8 (121.2)	.044
The deceased person was (*n* [%])				
Child or spouse	83 (15.3)	40 (10.3)	43 (28.3)	<.001
Parent or sibling	318 (58.7)	239 (61.3)	79 (52.0)	
Another family member	89 (16.4)	70 (17.9)	19 (12.5)	
Another person	52 (9.6)	41 (10.5)	11 (7.2)	
Cause of death (*n* [%])				
Unexpected (e.g., COVID-19, accident)	49 (9.0)	25 (6.4)	24 (15.8)	<.001
Expected (e.g., Alzheimer, cancer)	493 (91.0)	365 (93.6)	128 (84.2)	

TGI-SR, Traumatic Grief Inventory—Self Report.

### Measures

#### Risk Factors of Grief Reactions

*Common Risk Factors Related to the Bereaved, Deceased, and Death.* Participants were asked about their age, gender, marital status, household profile, and family income. The household profile was coded as living alone or not, no matter the person who lived with them. The age and gender of the deceased were also collected. The survey also included a question about the respondent's relationship with the deceased (*child or spouse; parent or sibling; another family member; another person*). This relation was recoded for correlational analyses as 0 = *other*; 1 = *immediate family* (i.e., *child* or *spouse* and *parent* or *sibling*). The time since death in days was calculated using the date of the death provided by the participant and the date of survey completion. Participants indicated the cause of death for the person the survey related to, and answers were coded as unexpected (including accident, suicide, COVID-19) and expected (including cancer, Alzheimer's disease, chronic diseases) causes.

*Specific Risk Factors Related to Restricted Funeral Rituals.* Participants were asked about their desire to be (or not) with the deceased at the end of life, if they were able to be there, and to what extent restrictions caused a gap between their desire and what happened (1 = *not at all* to 4 = *a lot*). A list of eight common funeral rituals was presented to participants and, for each ritual (e.g., *gatherings with relatives; touching or kissing the deceased person*), people were asked four questions: (a) Would you like the following ritual to be performed?, (b) Was the ritual performed?, (c) If not performed, was the ritual prevented due to health restrictions?, and (d) If performed, how satisfied are you with the ritual? (1 = *not at all satisfied*; to 4 = *totally satisfied*). The number of rituals wanted by participants and prevented by health restrictions was used as an indicator of objective restrictions in mourning rituals.

#### Mediators of Grief Reactions

The mean satisfaction regardine all rituals performed was used as a first mediator between risk factors and grief complications. The French version of the Bereavement Coping Scale was used to assess the oscillation process between two types of coping strategies (confrontation and avoidant; Delespaux et al., 2013) as well as the extent to which bereaved people used either confrontation or avoidant strategies. Based on the DPM (Stroebe & Schut, 1999, 2010), this scale includes 14 items related to loss-oriented stressors and 14 items related to restoration-oriented stressors. In each stressor category, seven items relate to confrontation strategies and seven to avoidant strategies. The scale shows good internal consistency in our sample (confrontation strategies: α = 0.83; avoidant strategies: α = 0.88). All items are scored on a 5-point Likert scale (0 = *almost never* [*less than once a month*] to 4 = *all the time* [*several times a day*]). The DPM assumes that two types of oscillation occur: one between loss- and restoration-oriented stressors or coping and one between confrontation and avoidant strategies (Stroebe & Schut, 2010). In the present study, the oscillation between strategy types, no matter the type of stressors, was used and integrated into the model. The mean score of the 14 items related to confrontation strategies was subtracted from the mean score of the 14 items related to avoidant strategies to obtain the oscillation score (Caserta et al., 2014; Delespaux et al., 2013). An oscillation score of 0 indicates a perfect balance between confrontation and avoidant strategies, while a negative score indicates that the person used a higher number of avoidant strategies than confrontation strategies. Additionally, to represent the extent of the use of coping strategies, the mean scores of both confrontation and avoidant strategies were calculated, leading to two scores of used strategies ranging from 0 to 4.

To assess the use of bereavement support services, participants were asked about telephone services (e.g., Tel-Aide, 1888-LE-DEUIL), online forums related to bereavement, and bereavement support groups (virtual or in-person). For each service, participants rated the frequency of their use (1 = *never*; 2 = *rarely*; 3 = *frequently*; 4 = *often*). The three values of frequency were summed up as an indicator of bereavement support.

#### Outcome: Grief Reactions and Complications

Grief reactions and complications were assessed by the 18-item version of the Traumatic Grief Inventory—Self Report (TGI-SR; Boelen & Smid, 2017). The French–Canadian version used in the present study showed evidence of internal consistency (α = 0.94) and construct validity (Cherblanc, Gagnon et al., 2022). Each item of the TGI-SR is scored on a 5-point Likert scale (1 = *never*, 5 = *always*) for a total score of 90, with a higher score indicating a higher level of grief reactions. A score ≥59 obtained more than 6 months after the death was used as an indicator of the presence of a possible PGD (Boelen et al., 2019).

### Ethical Considerations

Participation was voluntary; consent was obtained from each participant for the collection and analysis of their demographic information and patient-reported outcome measures. All surveyed participants were entered in a draw to win one of ten $20 gift cards. Ethics approval for the project (project #2021-697) was awarded by the Research Ethics Committee of the Université du Québec à Chicoutimi.

### Statistical Analysis

Considering the nonnormal distribution of continuous variables (age, days since loss, TGI-SR score), nonparametric statistical tests were chosen. Comparisons between those who were included and excluded in this analysis were made using the chi-square test (for gender) and Mann–Whitney U test (for age and number of days since loss). Sociodemographic and loss-related characteristics were compared between participant groups based on the cutoff score of the TGI-SR (<59 and ≥59) using the chi-square test for categorical variables and the Mann–Whitney U test for continuous variables. The total TGI-SR score was compared among participants according to their gender, household profile (alone or not), and cause of death (unexpected and expected causes) using the Mann–Whitney U test and among participants according to their relationship with the deceased person using the Kruskal–Wallis test. The proportion of PGD according to the participant's gender, household profile, relationship with the deceased, and cause of death was compared using the chi-square test.

**Figure 1.**
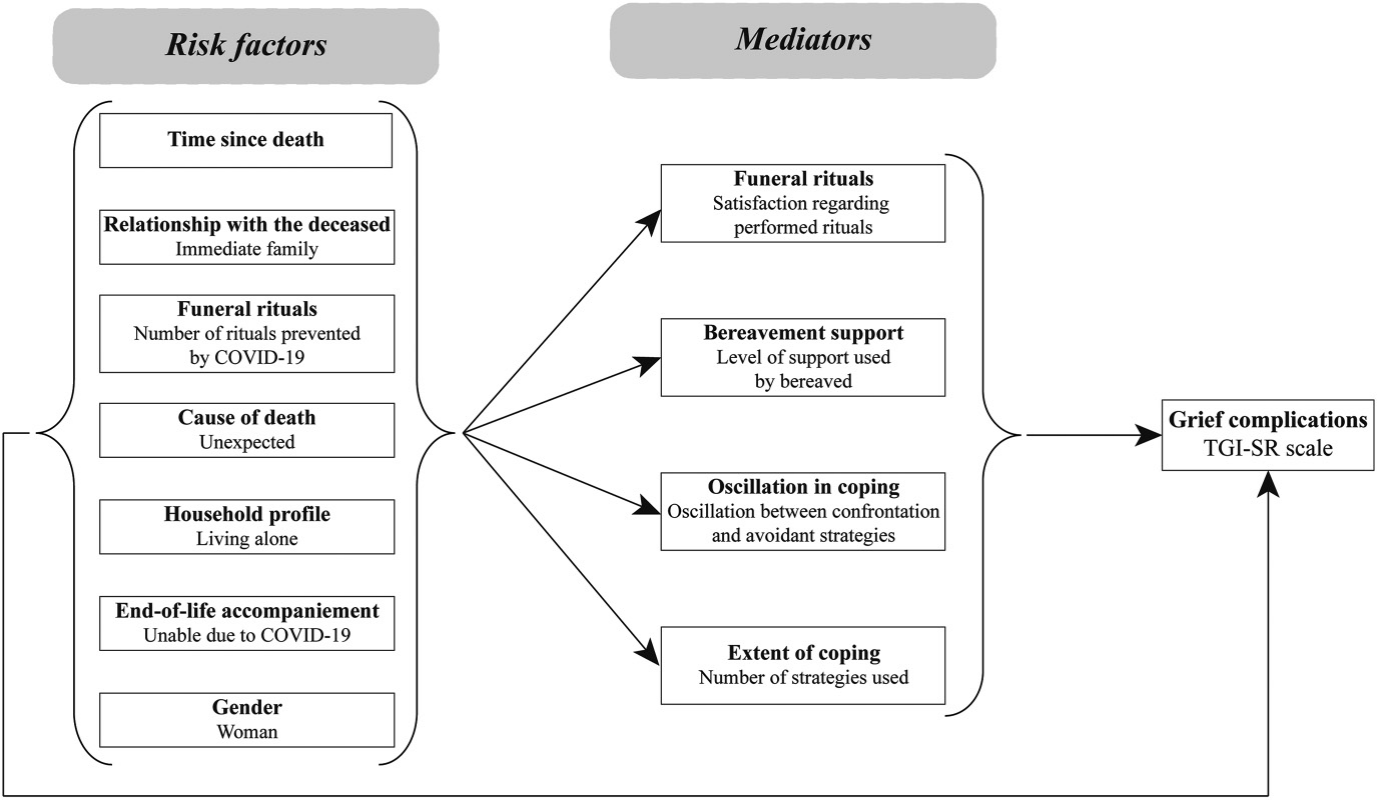
The theoretical model of grief complication predictors.

Based on the literature review, the expertise of the team members and the DPM, which suggests that coping strategies play a mediating role (Delespaux et al., 2013; Fasse & Zech, 2016), a theoretical model of grief was built before conducting analyses (see Figure 1). Associations between all variables included in the theoretical model were analyzed using Spearman's rho coefficient correlation. Data were analyzed using IBM SPSS Statistics for Windows, Version 28.0 (IBM Corp., Armonk, NY).

Structural equation modeling (SEM) was used to test the model of the mediating role of coping strategies on the relationship between death circumstances (including risk factors and sociodemographic characteristics) and PGD. Only variables that significantly correlated with the TGI-SR total score were included in the first model and tested using SEM analysis. The model was then modified and tested to obtain the best model fit using the following fit indices and cutoff criteria: ꭓ^2^/df ratio ≤2, Comparative Fit Index (CFI) > 0.95, Root Mean Square Error of Approximation (RMSEA) < 0.06, and Standardized Root Mean Squared Residual (SRMR) < 0.08 (Schreiber et al., 2006). The maximum likelihood estimation method was used given the number of missing values, and SEM analyses were performed using the CALIS procedure of SAS software (version 9.4).

Multiple regression analysis (R^2^) was performed with the variables included in the final model tested with SEM to assess how well they were able to predict the level of PGD as measured by the TGI-SR. Data were checked to ensure the normality of regression residuals (Kolmogorov–Smirnov test).

## Results

Of the 542 persons included in this study, approximately 32% qualified for PGD, that is, 93 out of the 290 people who had experienced death more than 6 months before the completion of the survey. The level of grief complications (TGI-SR total score) and the proportion of participants endorsing probable PGD among each of the categorical variables included in the theoretical model are shown in Table 2. Results show that the proportion of men and women between participants having or not a possible diagnosis of PGD (TGI-SR cutoff score ≥59) is similar. Consistent with previous results concerning risk factors, those living alone, having lost a child or spouse, and unexpected deaths presented higher scores and elevated probable prevalence of PGD as measured by the TGI-SR score.

**Table 2.** Correlational Analysis Between Sociodemographic or Death Characteristics and Grief Scores.

Characteristics	TGI-SR^1^ Total score (*n* = 542)	*p*-value	Prolonged grief disorder^2^		*p*-value
			Yes (*n* = 93)	No (*n* = 197)	
Participant's gender					
Men	46.2 (17.2)	.202	10 (10.8)	19 (9.6)	.769
Women	48.6 (14.9)		83 (89.2)	178 (90.4)	
Living alone					
Yes	54.3 (14.0)	<.001	37 (39.8)	42 (21.3)	<.001
No	46.4 (15.1)		56 (60.2)	155 (78.7)	
Deceased person was					
Child/spouse	58.3 (13.7)	<.001	24 (25.8)	19 (9.6)	.004
Parent/sibling	47.3 (14.8)		51 (54.8)	127 (64.5)	
Another family member	44.6 (15.2)		11 (11.8)	30 (15.2)	
Another person	45.0 (13.5)		7 (7.5)	21 (10.7)	
Cause of death					
Unexpected	56.4 (14.9)	<.001	15 (16.1)	11 (5.6)	.003
Expected	47.5 (15.0)		78 (83.9)	186 (94.4)	

*Note*. 1. Results are presented as the mean (standard deviation). The *Traumatic Grief Inventory—Self Report (TGI-SR*) total score was compared between participant characteristics using the Mann–Whitney U test or Kruskal–Wallis test. 2. TGI-SR score ≥59 and time since loss > 6 months for prolonged grief disorder diagnosis. The results are presented as *n* (% within a category). The proportion of prolonged grief disorder was compared using the chi-square test.

**Figure 2.**
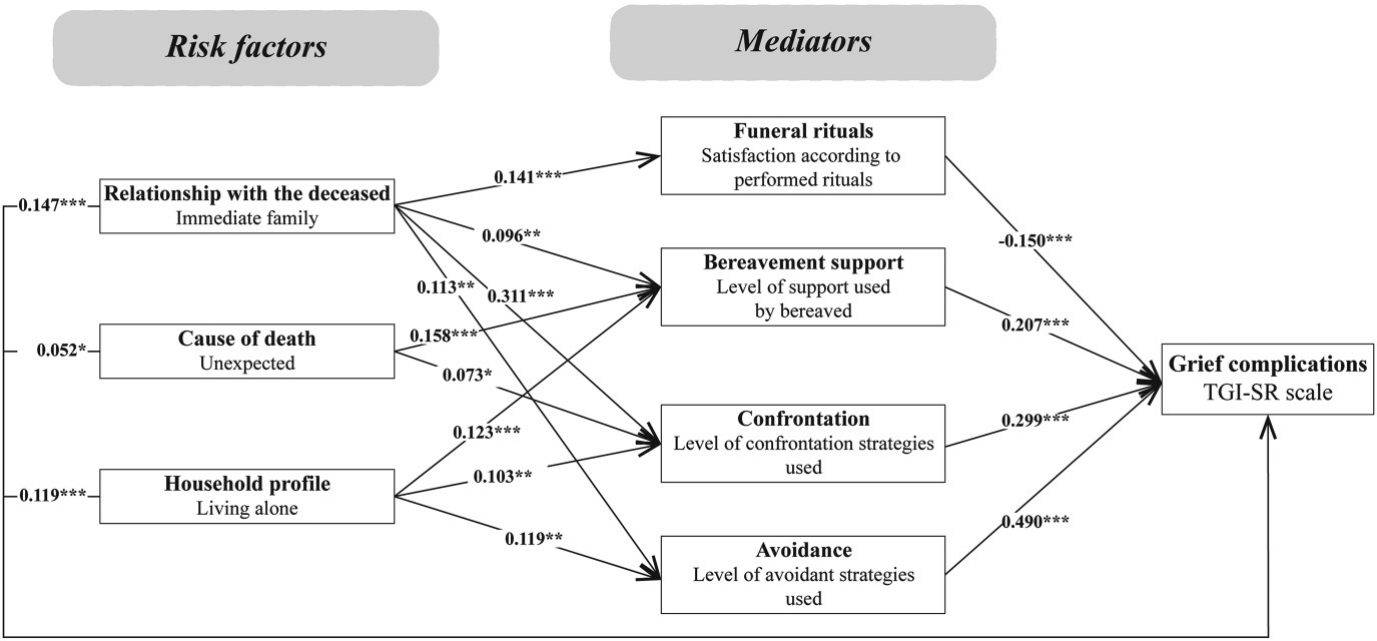
Initial model. Fit indices of the SEM analysis: ꭓ^2^/df = 8.9; CFI = 0.8873; RMSEA = 0.0945; SRMR = 0.0564. ***p < .001 **p < .01 *p < .05.

*Note.* SEM = structural equation modeling; CFI = Comparative Fit Index; RMSEA = Root Mean Square Error of Approximation; SRMR = Standardized Root Mean Squared Residual.

The variables included in the theoretical model (see Figure 1), were tested for their correlations with the TGI-SR. As seen in Table 3, eight variables were significantly associated with a higher level of PGD. In addition, contrary to what was expected, the time since the loss, participant's gender, and being unable to be present with the dying person at the death were unrelated to grief reactions. It should be noted that the oscillation between confrontation and avoidant coping strategies was the only mediator not significantly related to the TGI-SR. Considering the low correlation with the TGI-SR and the collinearity with the satisfaction regarding performed rituals, the number of rituals was not included as an independent variable in the SEM analyses. The other seven variables (three risk factors and four mediators) were kept for the initial model tested using SEM analysis (see Figure 2).

**Table 3.** Correlations Between the Traumatic Grief Inventory—Self Report (TGI-SR) Score and the Variables That Relate to Coping Strategies.

	Spearman rho correlation coefficients											
	1.	2.	3.	4.	5.	6.	7.	8.	9.	10.	11.	12.
1. TGI-SR	–											
2. Days since loss	0.05	–										
3. Relationship	0.14**	0.07	–									
4. Rituals	0.10*	0.09*	−0.05	–								
5. Cause	0.16**	0.004	−0.14**	−0.09*	–							
6. Living alone	0.22**	0.08	0.08	0.005	−0.002	–						
7. Presence	0.07	0.07	0.04	0.37**	−0.21**	−0.006	–					
8. Gender	−0.06	−0.06	0.09*	0.06	0.04	0.06	0.06	–				
8. Satisfaction	−0.16**	−0.17**	0.12**	−0.59**	0.006	−0.01	−0.28**	−0.06	–			
10. Support	0.34**	0.06	0.11*	−0.08	0.20**	0.17**	0.03	−0.04	0.09*	–		
11. Oscillation	−0.04	0.008	0.15**	−0.003	0.05	0.02	0.03	−0.03	0.04	0.07	–	
12. Confrontation	0.39**	0.009	0.28**	0.02	0.03	0.14**	0.07	−0.04	0.01	0.23**	0.69**	–
13. Avoidant	0.56**	0.03	0.11*	0.02	−0.03	0.14**	0.03	−0.04	−0.07	0.17**	−0.52**	0.19**

*Note*. 1. Traumatic Grief Inventory—Self-Report total score; 2. Number of days since the death occurred; 3. Relationship with the deceased person (0 = *other person*, 1 = *immediate family*); 4. Number of rituals wanted by participants and prevented by COVID-19; 5. Cause of death (0 = *expected*, 1 = *unexpected*); 6. Household profile (0 = *live with other people*, 1 = *live alone*); 7. Being unable to be present with the dying person due to COVID-19; 8. Gender (0 = *men*, 1 = *women*); 9. Satisfaction regarding performed rituals; 10. The extent of bereavement support the participant looked for; 11. The oscillation between confrontation and avoidant strategies used; 12. Mean score of confrontation strategies; 13. Mean score of avoidant strategies.

*p < .05 **p < .001.

**Figure 3.**
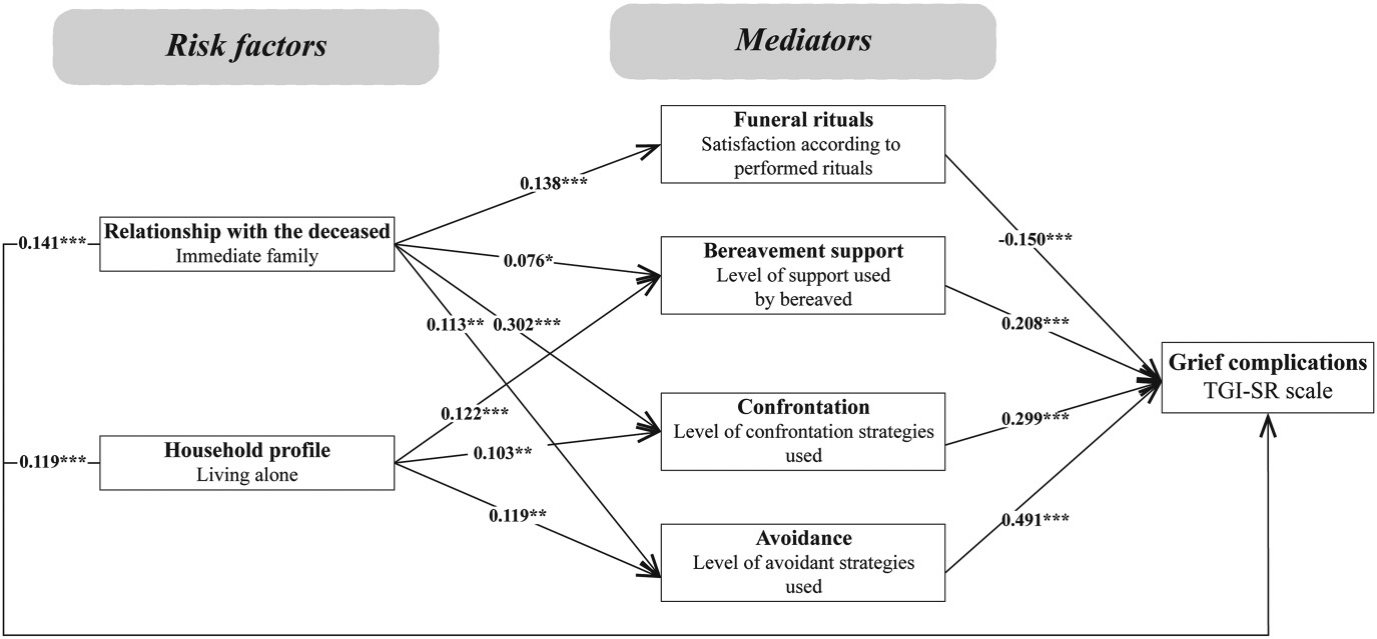
The best model was selected after model reduction. Fit indices of the SEM analysis: ^ꭓ2^/df = 7.1; CFI = 0.9147; RMSEA = 0.0836; SRMR = 0.0608. ***p < .001 **p < .01 *p < .05.

*Note.* SEM = structural equation modeling; CFI = Comparative Fit Index; RMSEA = Root Mean Square Error of Approximation; SRMR = Standardized Root Mean Squared Residual.

None of the models tested obtained excellent fit indices. The most parsimonious model having the best-fit indices is presented in Figure 3. In this model, an adequate but not excellent adjustment of the data was obtained. Only one index reached the desired cutoff (SRMR = 0.0608) while CFI and RMSEA approached the cutoff criteria (0.9147 and 0.0836, respectively). The results show that all variables directly influenced the level of grief complications as measured by the TGI-SR. Coping strategies had a significant mediating role regarding grief complications. Being bereaved by the death of someone from the immediate family was associated with a higher level of coping strategies used (both confrontation and avoidant), which was associated with higher grief complications. Also, a lower level of satisfaction regarding the rituals played a mediating role between the loss of a kin/close family member and grief complications. Living alone was associated with a higher use of bereavement support as well as a higher level of confrontation and avoidant coping strategies used. Finally, the level of avoidant strategies used was the variable having the strongest path in the model. The more avoidant strategies are used, the more complicated grief reactions are. This positive relationship was also present between confrontation strategies and grief reactions.

The extent of variance of the TGI-SR explained by the variables included in the best model was tested with a linear multiple regression analysis. Although this type of analysis does not allow testing for a mediator effect on a variable, it permits a better understanding of how much the predictors explain the variance of the dependent variable. The regression analysis was performed in two steps: first, risk factors were entered into the model and then, mediators were added to see the effects on the R^2^ (see Table 4). While risk factors explained only 6.6% of the total variance of the TGI-SR, the addition of mediators increased to 44.4% the level of explanation of the variance of grief complications. As for the SEM, the level of avoidant strategies used was the variable that most explained the level of grief complications.

**Table 4.** Multiple Linear Regression Analysis Results Using ENTER Method with the TGI-SR Total Score as the Dependent variable.

R^2^	F-value	*p*-value	Independent variables	β	95% CI	β_std_	*p*-value	VIF
*Risk factors only*								
0.066	19.104	<.001	Constant	43.30	40.80–45.79	—	<.001	—
			Household profile	7.65	4.76–10.53	0.217	<.001	1.006
			Relationship with the deceased	4.23	1.40–7.07	0.122	.004	1.006
*With coping mediators*								
0.466	77.955	<.001	Constant	22.42	17.76–27.09	-	<.001	-
			Household profile	3.38	1.14–5.62	0.096	.003	1.050
			Relationship with the deceased	0.237	−2.03–2.50	0.007	.837	1.113
			Funeral rituals—satisfaction	−2.66	−3.80–−1.51	−0.147	<.001	1.033
			Bereavement support	2.80	1.90–3.69	0.201	<.001	1.080
			Confrontation strategies	5.50	4.09–6.90	0.261	<.001	1.163
			Avoidant strategies	10.79	9.27–12.31	0.454	<.001	1.064
			Residual normality	*p* = .200				

*Note.* Categorical variables are coded as follows: Household profile: 0 = *live with other people*, 1 = *live alone*; Relationship with the deceased: 0 = *another person*, 1 = child/spouse/parent/sibling.

TGI-SR, Traumatic Grief Inventory—Self Report.

## Discussion

This study tested a model of grief complications integrating both risk factors and mediators in the context of the COVID-19 pandemic. It provides important insights into the experience of bereavement in several ways. First, it shows a notably high prevalence of possible PGD in 33.3% of respondents having become bereaved during the COVID-19 pandemic for more than 6 months before being surveyed. This prevalence is three times more than what can be found in general studies (Lundorff et al., 2017), but lower than recent studies also conducted during the COVID-19 pandemic (Bovero et al., 2022; Breen et al., 2022; Lee & Neimeyer, 2022).

Second, this study is one of the first to include mediating factors in addition to risk factors. As recommended by Stroebe and Schut (2021), we tested a theoretical model that included how bereaved people experienced and dealt with the death of the person rather than only objective circumstances that are intrinsic to their situation or imposed (e.g., public health restrictions to rituals). From a political, ill-health preventive, and sociological point of view, it is important to identify objective circumstances and risk factors that may be associated with detrimental ill-health or PGD (e.g., living alone, imposed public health restrictions to save lives). However, bereaved people have little or no control over these factors. Recommendations for helping the bereaved may rather address and focus on the amenable aspects of the grieving processes and thus on the mediators of grief reactions. Our results showed that the way bereaved people are satisfied with funeral rituals, how they search for and find support, and to what extent they use both confrontation and avoidant coping strategies better explain how they will experience their grief. While avoidant strategies were the strongest direct predictor of grief reactions in the regression analysis, confrontation strategies were the strongest mediator between the relationship with the deceased and living alone and grief reactions.

Regarding the relationship with the deceased, our results show a significant association with grief reactions, that is mediated by all mediators. This is very consistent with the theoretical and empirical literature: the kinship relationship to the person is a major predictor of grief reactions, explaining more than other known predictors such as gender, time since loss or death circumstances (Fernández-Alcántara & Zech, 2017). Consistently, while the initial model tested using SEM included the unexpected cause of death as a risk factor—a result that is well known and previously found in the current pandemic context as a predictor of grief complications (e.g., Eisma et al., 2021)—the final model did not retain the death circumstances as a predictor and the relationship with the deceased remained the strongest predictor in the model. We speculate that no matter the cause of the death, the COVID-19 pandemic may have “homogenized” the context surrounding the death by making uncertainty or unexpectancy overly prevalent. For example, even in the case of expected death, many people were unsure under which conditions they could accompany their relative at the time of death, perform rituals, meet other ones, or even go (or not) back to work or contract the COVID and die. In addition, the present study confirmed that being an immediate family member was a predictor of mediators such as experiencing more satisfaction with the rituals that were performed. This is probably because bereaved parents and widowed people were those who could be the ones choosing the (few) funeral rites that could finally be performed for their child or partner, even in times where 66.2% of these rites could not be performed as desired (Cherblanc, Zech et al., 2022). Interestingly, and for the first time in the literature, the results of the present study showed that being a close family member of the deceased was a predictor of using more coping strategies, especially more confrontation than avoidant coping strategies. Contrary to a recent study (Zou et al., 2022), our analysis shows that avoidance *and* confrontation strategies, rather than avoidance (or confrontation) only, mediated the association between risk factors and grief complications. The explanation of our unique finding may pertain to the deprivation of rituals in the pandemic context. Since rituals have the function of acting out death (Thomas, 1985, 2003), they may constitute strategies of intense coping strategies. The inability to hold rituals because of public health measures might have had the consequence to distribute both coping strategies in space and time and increase their number as a kind of compensation. This may be particularly the case among people experiencing grief complications. This interpretation is in line with the finding that bereaved people who were living alone have looked for more bereavement support than those who were living in couples, with children, or with other people. This well-known risk factor of grief severity was also associated with more intensive use of coping strategies (regardless of type).

This study is innovative and original in testing whether the rituals before, during, and after death, as well as the expected relationship between being unable to attend, care, be present or say goodbye when the close person was dying, would predict more severe grief reactions and PGD prevalence. Contrary to what was mostly predicted in the early literature on grief in times of COVID (e.g., Doka, 2021; Eisma et al., 2021), we did not find direct relationships between the number of prevented rituals and grief outcomes. This nonsignificant result mirrors the results of a recent study (Mitima-Verloop et al., 2021). However, as explained before, satisfaction with rituals was a good mediator of grief reactions.

Finally, we want to conclude with a result that was contrary to what is usually expected when one says “*time heals all wounds*”: in the present study, the delay since the death occurred was unrelated to grief severity. In the literature, the effect of time seems to be inconclusive, since some authors found a correlation with probable PGD (Gang et al., 2022) while others did not (e.g., Lee et al., 2021). A potential explanation of this non-significant result may be that the elapsed delay since bereavement (a maximum of just over a year after the beginning of the pandemic) was too short for many of the respondents, or that the pandemic context, which was still going on during the survey, may have represented an “overload” of stressors (Stroebe & Schut, 2016), and thus had a negative impact on grief reactions, at least for part of our sample, thereby leading to nonsignificant results.

### Limitations

Although the present study tested a comprehensive model of coping with bereavement in times of the COVID-19 pandemic, including risk and protective factors, mediators, and grief outcomes, it should be stressed that, for now, we only tested it in a cross-sectional way. As such, we cannot assert that perceived dissatisfaction, coping, and support services are truly mediating factors or even that grief reactions are outcomes rather than predictors. Coping and support strategies might likewise be considered co-occurring with the other measures taken at the same moment of data collection. For example, in our tested model, reaching out to bereavement support services was considered a mediator and was found to be positively related to higher grief reactions. This result would suggest that contrary to the literature (Judah & Jeanne, 2016; Robinson & Pond, 2019), the use of these services for the bereaved should be avoided, or that they are not sufficient (Aoun et al., 2012). However, an alternative more likely hypothesis is that when bereaved people experience very high and distressing grief reactions, they will try to cope with them and thus search for more support services to share, understand, and validate their personal experiences (Judah & Jeanne, 2016; Swartwood et al., 2011). We cannot yet answer whether the use of support systems or confrontation rather than avoidant strategies will help bereaved individuals cope with their grief. To address mediating processes more fully, the study design should include longitudinal assessments to track changes over time and distinguish between moderators and mediators of grief outcomes (Lechner-Meichsner et al., 2022). The exploration of how bereaved individuals experienced the restrictions of pre- and postdeath rituals also needs further exploration, which involves the collection of qualitative data. For these reasons, we will follow the same sample of bereaved people during the next 2 years with quantitative online surveys every 6 months as well as conducting interviews among a subsample of participants. This may allow us to answer some of the questions this model raises.

It should also be acknowledged that our sample was self-selected since our participants were reached through letters from funeral homes and advertisements in social and public media. It is likely that those most interested in bereavement or reaching out for help were also those experiencing (or at potentially elevated risk of) more severe and disrupted grief reactions. This might lead to an overestimation of probable PGD prevalence and a bias in how bereaved people experienced the grieving processes in comparison to a community-based sample that is recruited only through death certificates or obituaries. The presence of this bias tends to be confirmed by the result that those searching for support on websites or in bereavement groups were also more likely to present with higher grief reactions in the present sample.

### Final Considerations

The present study highlights the importance to distinguish between risk and mediating factors to explain why bereaved individuals will report and experience severe grief reactions or not. It showed that mediators such as being unsatisfied with funeral rituals, searching for bereavement support, or both confrontation and avoidant coping strategies, are better explanatory variables than well-known risk factors. The context of the pandemic, having caused major changes in people's lives, their well-being, and an increased number of deaths, has led to a unique opportunity to examine grief processes under unprecedented conditions of restrictions to rituals. This allows for an exploration of the a priori assumptions that performing mourning rituals lead to less severe or less complicated grief reactions.
